# Brassinosteroids, the Sixth Class of Phytohormones: A Molecular View from the Discovery to Hormonal Interactions in Plant Development and Stress Adaptation

**DOI:** 10.3390/ijms20020331

**Published:** 2019-01-15

**Authors:** Ana Laura G. L. Peres, José Sérgio Soares, Rafael G. Tavares, Germanna Righetto, Marco A. T. Zullo, N. Bhushan Mandava, Marcelo Menossi

**Affiliations:** 1 Functional Genome Laboratory, Department of Genetics, Evolution, Microbiology and Immunology, Institute of Biology, State University of Campinas, Campinas 13083-970, Brazil; analaura@lgf.ib.unicamp.br (A.L.G.L.P.); zesergio@lgf.ib.unicamp.br (J.S.S.); germanna@lgf.ib.unicamp.br (G.R.); 2 Center for Tropical Crops and Biocommodities, Queensland University of Technology, Brisbane, QLD 400, Australia; rafael@lgf.ib.unicamp.br; 3 Laboratory of Phytochemistry, Agronomic Institute, Campinas 13020-902, Brazil; mzullo@uol.com.br; 4 Mandava Associates, LLC, 1050 Connecticut Avenue, N.W. Suite 500, Washington, DC 20036, USA; bhushan.mandava@verizon.net

**Keywords:** brassinosteroids, plant hormones, hormonal cross-talk

## Abstract

Phytohormones are natural chemical messengers that play critical roles in the regulation of plant growth and development as well as responses to biotic and abiotic stress factors, maintaining plant homeostasis, and allowing adaptation to environmental changes. The discovery of a new class of phytohormones, the brassinosteroids (BRs), almost 40 years ago opened a new era for the studies of plant growth and development and introduced new perspectives in the regulation of agronomic traits through their use in agriculture. BRs are a group of hormones with significant growth regulatory activity that act independently and in conjunction with other phytohormones to control different BR-regulated activities. Genetic and molecular research has increased our understanding of how BRs and their cross-talk with other phytohormones control several physiological and developmental processes. The present article provides an overview of BRs’ discovery as well as recent findings on their interactions with other phytohormones at the transcriptional and post-transcriptional levels, in addition to clarifying how their network works to modulate plant growth, development, and responses to biotic and abiotic stresses.

## 1. Introduction

**Figure 1:**
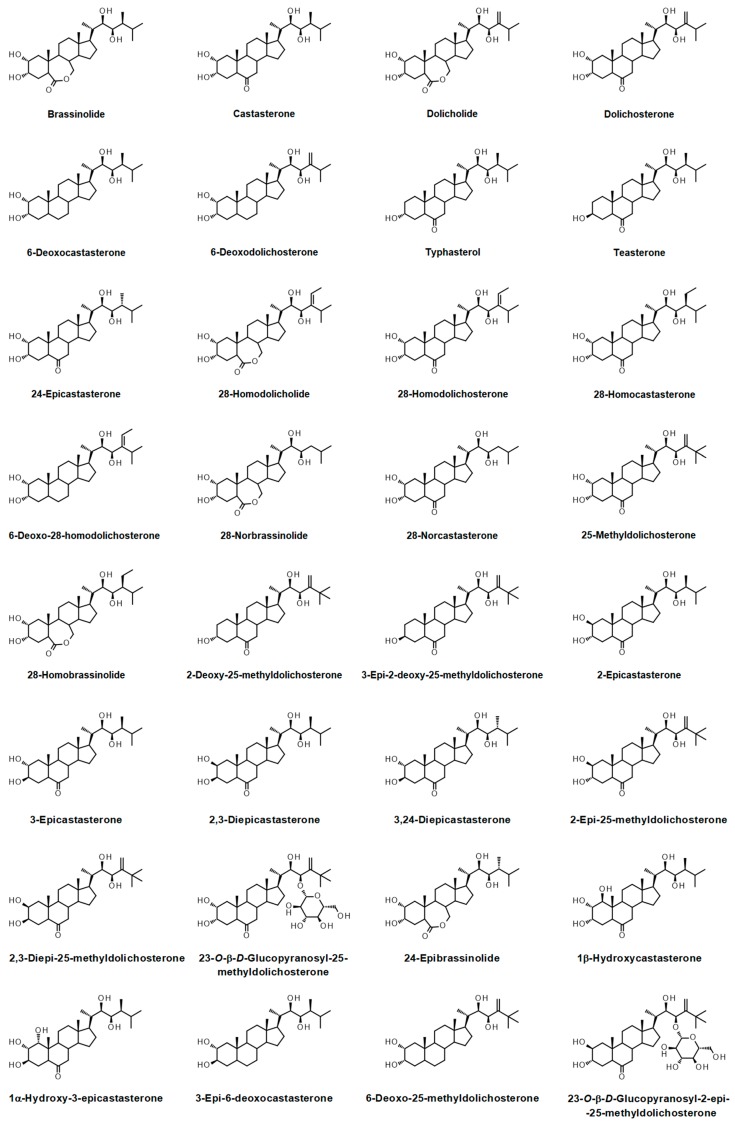

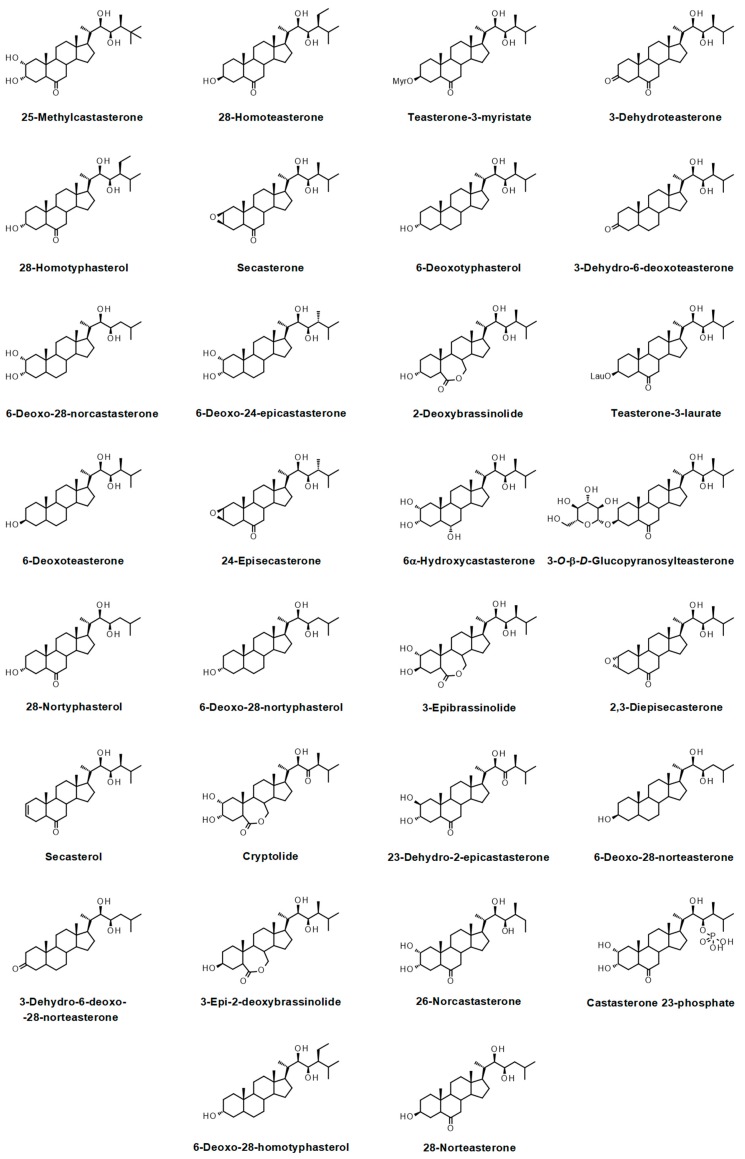
Natural brassinosteroids isolated from or detected in plant sources.

In the first years of the 20th Century, the only known plant hormones with recognized roles in development were indole-acetic acid and gibberellic acid. Some early experiments demonstrated that the application of the spores or pollen of some plants to the stigmas of other species promotes the development of parthenocarpic fruits therein. Even pollen extracts [1], and some growth-promoting chemicals [2], were shown to promote parthenocarpy. When applied to the first internode of intact bean plants, ethereal extracts from corn pollen caused pronounced elongation compared to control and even plants treated with natural or synthetic auxins [3]. The same effect was obtained with extracts prepared from immature bean seeds [4]. It was further shown that *Brassica napus* and *Alnus glutinosa* pollens contain some plant growth regulators, termed brassins, considered plant hormones as they were supposed to be “specific translocatable organic compounds isolated from a plant and have induced measurable growth control when applied in minute amounts to another plant” [5]. The pollen extracts of many other plant species showed the same effects [6], but it was not possible at that time to attribute the physiological effects observed by the application of brassins to any known compound. After a time-consuming and expensive multidisciplinary effort [7]—that involved the processing of at least 400 pounds of rape pollen by a newly-developed method for obtaining brassins [8] as well as physiological and agronomical assays with the active fractions—brassinolide (BL) (Figure 1) [9] was identified as the compound responsible for the different physiological effects produced by brassins. The first syntheses of BL [10,11] and similar compounds [12,13,14,15] were soon reported, and the development of a micromethod for their detection [16] (from which many others derived [17,18]) revealed compounds resembling BL in many plant species. In the coming years the isolation of many other compounds with structures similar to BL gave rise to the family of brassinosteroids (BRs) [19,20,21,22,23,24], defined as the “3-oxygenated (20β)-5α-cholestane-22α,23α-diols or their derived compounds isolated from plants, bearing additional alkyl or oxy substituents” [25], now recognized as the sixth class of plant hormones. This class of phytohormones is represented by more than 60 compounds (Figure 1) that have been isolated or detected from more than 100 plant species, from algae to angiosperms, revealing their ubiquitous distribution in the plant kingdom [25,26].

Simultaneous to efforts being made to isolate the active principle(s) of the brassins, experiments were being conducted to verify their possible beneficial effects on crops [27,28] as well as to determine their hormonal functions [29,30,31]. The first syntheses of BL [10,11], 28-homobrassinolide [12], 24-epibrassinolide [13], and other BRs allowed pure compounds to be assayed by the methods used for testing other established plant hormones, such as auxins [32,33], cytokinin, and gibberellin [34,35]. It also allowed their interactions with other plant hormones to be tested [36,37], providing a solid basis for understanding their actions in plant growth and development [38,39,40,41], including the role of BL in the germination and growth of pollen tubes [42]. Molecular analyses of BRs’ action soon appeared [43], and the discovery of BR-deficient mutants [44], BR-signaling mutants [45], and of BR biosynthesis inhibitors [46,47] made it possible to further determine their mechanisms of action at the molecular level. The elucidation of the BL structure and its receptor kinase BRASSINOSTEROID INSENSITIVE 1 (BRI1) provided insight into the recognition of BRs by their receptor and the activation of the BL-BRI1 complex [48,49]. The evolution of the research into the physiological and biochemical aspects of brassinosteroids is reviewed elsewhere [50].

Previous and recent studies have indicated how the cross-talk between BRs and other phytohormones might contribute to the regulation of an extensive spectrum of biological processes. The present review provides an overview of the current knowledge on the cross-talk between brassinosteroids and other phytohormones, such as auxin (AUX), gibberellins (GAs), cytokinins (CKs), ethylene (ET), abscisic acid (ABA), jasmonic acid (JA), and salicylic acid (SA) at the transcriptional and post-transcriptional levels, as well as how their networks may contribute to the modulation of plant growth, development, and other biological processes. Our major objective is to provide a clear understanding of how BR in conjunction with other phytohormones controls different activities in plant metabolism.

## 2. Brassinosteroids: Functions and Signaling Pathway

Due to BRs’ growth regulator activity, this class of phytohormones is involved in a range of developmental processes, including cell division and elongation, vascular differentiation, reproductive development and the modulation of gene expression [51]. BR-deficient and -insensitive mutants in *Arabidopsis thaliana* (hereafter called *Arabidopsis*) present dwarfism, short petioles, delayed flowering, and reduction in fertility phenotypes. Equivalent mutants in other eudicot species such as tomato (*Solanum lycopersicum*), pea (*Pisum sativum*), and petunia (*Petunia hybrida*), as well as in monocots, like rice (*Oryza sativa*), barley (*Hordeum vulgare*), and maize (*Zea mays*) showed comparable phenotypes [52,53,54].

The main responsible for BR-mediated responses are BZR1 (BRASSINAZOLE RESISTANT 1) and BES1 (BRI1-EMS SUPPRESSOR 1), also named BZR2, the two major BR signaling pathway transcription factors, which regulate a range of genes involved in different physiological processes, such as developmental responses, protein metabolism, cellular transport and signaling, cell wall biosynthesis, chromatin and cytoskeleton components, environmental responses, and hormone responses [55].

### Signaling Pathway

In previous years, a combination of genetic, biochemical and proteomic approaches have accelerated the understanding of the BR signaling pathway in *Arabidopsis* [52,56,57,58]. Upon BR binding, BRI1 (BRASSINOSTEROID INSENSITIVE 1), a plasmatic membrane leucine-rich repeat (LRR) receptor-like kinase (RLK) [59,60], which functions with its coreceptor BAK1 (BRI1-ASSOCIATED RECEPTOR KINASE 1) [61,62,63], generates a phosphorylation cascade [64,65]. Activation of the receptor and coreceptor stimulates the phosphorylation of BKI1, the inhibitor of BRI1 [66,67], leading to its dissociation from the plasma membrane and further association with 14-3-3 proteins. The 14-3-3 proteins are involved in the interaction and cytoplasmic retention of BZR1 and BES1 [68,69,70,71,72]. Concomitantly, activated BRI1 is also involved in the phosphorylation of the BSKs (BR-SIGNALING KINASE 1) and CDG1 (CONSTITUTIVE DIFFERENTIAL GROWTH 1), which both subsequently activate BSU1 phosphatase (BRI1 SUPPRESSOR 1) [57,73,74,75]. BSU1 is responsible for dephosphorylating BIN2 (BRASSINOSTEROID-INSENSITIVE 2), a GSK3-like kinase and the major repressor of the BR signaling pathway [72], which is posteriorly repressed by KIB1 (KINK SUPPRESSED IN BZR1-1D), an F-box ubiquitin ligase that does not allow the association of BIN2 with BZR1/BES1, culminating in its ubiquitination and degradation [76]. Upon BIN2 inactivation, BZR1 and BES1 are rapidly dephosphorylated by PP2A (PHOSPHATASE 2A) and subsequently dissociated from 14-3-3 proteins, causing them to accumulate into the nucleus, resulting in the regulation of many BR-responsive genes [77].

**Figure 2:**
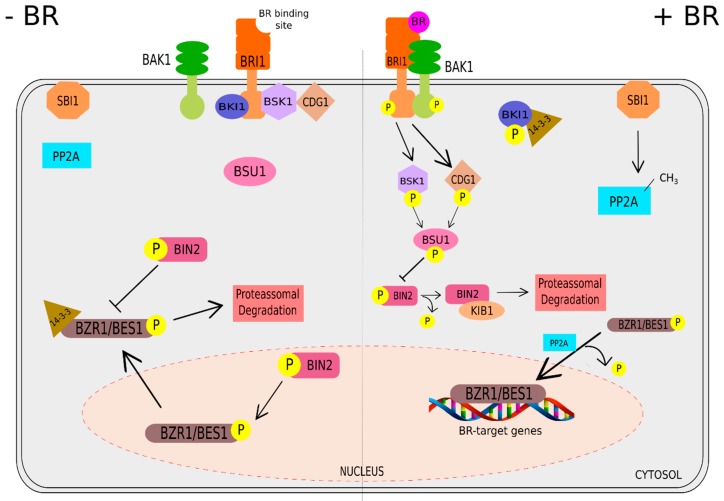
Current model of the signaling pathway in the presence or absence of brassinosteroids (BRs) in *Arabidopsis*. In the absence of BR, the receptor kinase BRI1 (BRASSINOSTEROID INSENSITIVE 1) does not heterodimerize with its coreceptor BAK1 (BRI1-ASSOCIATED RECEPTOR KINASE 1), maintaining their inactive forms. Consequently, BIN2 (BRASSINOSTEROID-INSENSITIVE 2), a negative regulator of BR signaling pathway, is free to constitutively phosphorylate BZR1 (BRASSINAZOLE RESISTANT 1) and BES1 (BRI1-EMS SUPPRESSOR 1), the two master transcription factors of BR-induced responses, inducing their interactions with 14-3-3 proteins that, in turn, promotes the cytoplasmic retention of BZR1/BES1, suppressing their DNA-binding activity. On the other hand, in the presence of BR, the activation of BRI1 triggers its autophosphorylation and partial kinase activity and dissociation from its inhibitor BKI1, which is attached at the BRI1 kinase domain. This leads to its heterodimerization with BAK1, and transphosphorylation to complete BRI1 kinase activity. Activated BRI1 then phosphorylates BSKs (BR-SIGNALING KINASES) and CDG1 (CONSTITUTIVE DIFFERENTIAL GROWTH 1) which both phosphorylate BSU1 (BRI1 SUPPRESSOR 1), leading to BIN2 dephosphorylation. BIN2 is subsequently restrained by KIB1 (KINK SUPPRESSED IN BZR1-1D), which prevents the association of BIN2 with BZR1/BES1 and facilitates its ubiquitination and degradation. The inactivated form of BIN2 allows BZR1 and BES1 to enter into the nucleus and regulate the expression of BR target genes. Additionally, PP2A (PHOSPHATASE 2A) also positive regulates BR signaling by dephosphorylating BZR1 and BES1, whereas SBI1 (SUPPRESSOR OF BRI1) deactivates BRI1 through the methylation of PP2A.

In the absence of BR, BKI1 binds to the intracellular domain of BRI1, preventing its association with its coreceptor BAK1 [66]. In turn, BIN2 is activated, and 14-3-3 proteins are associated with BZR1 and BES1, maintaining their dephosphorylated form and blocking their capability of shuttling to the nucleus for the regulation of thousands of BR responsive genes [67]. It is worth mentioning that previous studies have indicated that BR increases the expression of SBI1 (SUPPRESSOR OF BRI1), a positive regulator of BR1 degradation that methylates PP2A and controls its membrane-associated subcellular localization. As such, the relocation of methylated PP2A at membranes facilitates its association with the BR-activated BRI1, leading to BRI1 dephosphorylation and degradation, and, in turn, the termination of BR signaling. These data indicate that PP2A and SBI1 provide a negative feedback mechanism that triggers BRI1 turnover after activation of the BR signaling pathway [78]. The current model of the BR signaling pathway can be observed in Figure 2.

## 3. Cross-talk between BRs and Other Phytohormones in Plant Growth, Development, and Stress Responses

### 3.1. Brassinosteroids and Auxins

The events along the plant life cycle rely on coordinated changes at the molecular level in plant growth in a complex network, requiring a synchronism involving different hormone signals. Over the years, BR and auxin have been considered as two important phytohormones that function as master regulators in different plant development processes such as root development and stem elongation [79,80].

The interaction between BR and auxin has been observed in different processes. Hypocotyl elongation assays showed that auxin-responsive mutants display reduced BR sensitivity [81]. Similarly, BR treatment significantly enhanced auxin response in hypocotyl elongation, indicating that the auxin response depends on the presence of a functional BR signal transduction pathway [82].

Similar to BR, auxin is a growth-promoting hormone that is synthesized mostly in the shoot apical meristem (SAM), young leaves and in the root along the meristem [83,84] that binds to the TRANSPORT INHIBITOR RESPONSE1/AUXIN SIGNALING F-BOX (TIR1/AFB) receptor protein, which triggers the degradation of the AUXIN/INDOLE ACETIC ACID (AUX/IAA) transcriptional repressor protein. Upon ubiquitylation and subsequent degradation of the Aux/IAA proteins, AUXIN RESPONSE FACTOR (ARF), a family of transcription factors, including 25 and 23 members in rice and *Arabidopsis*, respectively, are released to activate gene expression through the recognition of auxin-responsive DNA Elements (AuxREs) [85,86,87]. The balance between AUX/IAA and ARF is a key control point in auxin signaling and orchestrates the molecular mechanisms by which auxin-BR impacts plant growth and development [88]. Besides, dual roles have been reported for ARFs: transcriptional activation and repression of gene expression.

The first molecular evidence of transcriptional regulation of ARF genes by BR came from the downregulation of *ARF4* and *ARF8* in BL-treated hypocotyls of *Arabidopsis* wild-type (WT) seedlings, contrasting the high level of expression observed in BR-deficient mutants [89]. In another study, the overexpression of *ARF8* in *Arabidopsis* inhibited the hypocotyl growth and resulted in a weaker apical dominance [90]. These results indicate that *ARF8* negatively regulates the auxin response in shoot elongation. The transcriptional activation activity of *ARF* was observed by chromatin immunoprecipitation-sequencing (ChiP-seq) and transgenic analyses where the interaction between BZR1 and ARF6 enhanced their DNA-binding activity capacity and promoted the activation of shared-target genes involved in hypocotyl elongation [91,92]. In addition, the ChIP assay confirmed that BZR1 binds to *IAA19* and *ARF7* promoters to potentiate the auxin response [93]. Interestingly, the application of high concentrations of BL or the hypersensitive *bzr1-1D* mutant resulted in curved and shorter hypocotyls [94]. All of these results indicate that BZR1 and an appropriate BR concentration are required for the auxin promotion of hypocotyl elongation in *Arabidopsis* seedlings grown in the dark. On the other hand, at low BR levels, another component of the BR signaling pathway, BIN2, phosphorylates ARF7 and ARF19, enhancing their DNA-binding capacity during lateral root development [95]. This corroborates with the inhibition of root growth by high levels of BR [96]. Nevertheless, the BIN2-mediated phosphorylation of ARF2 in the gain-of-function *bin2* mutant was shown to reduce ARF2 DNA-binding and its repressing activity on shoot and root growth [82]. These results are a clear indication that the auxin-BR response involves a dynamic coordination of both transcriptional and post-transcriptional regulation of ARFs via BZR1 and BIN2 to control plant growth and development in a spatiotemporal context.

Root development is determined by the balance between cell division and differentiation in the root meristem. Despite the well-known synergistic interaction in various developmental processes, in the case of root tips, BR and auxin interact antagonistically in controlling gene expression, stem cell maintenance and cell elongation. Additionally, a finely balanced concentration between these hormones is required for optimal root growth [97]. BR affects root growth in a concentration-dependent manner to control the root meristem size. The short root phenotype of the BR-insensitive *bri1-116* mutant is suppressed by low concentrations of BL [98]. Additionally, specific cell types of the root meristem are affected by different levels of BR. Chaiwanon et al. (2015) [97] observed that the expression of *bzr1-1D* in the *bri1-116* mutant epidermis cells increased the elongation zone of the root meristem. On the other hand, high levels of BR/BZR1 in the endodermis or in the quiescence center (QC) had no effect on the *bri1-116* phenotype, indicating the requirement of different concentrations of BR/BZR1 for the normal function of root cells [97]. Collectively, these observations support a model whereby, under different levels of BR, BZR1 contributes to the gene expression pattern by targeting different genes in distinct cells, as is the case in the induction of genes expressed in the transition-elongation zone, but repressing genes in the QC and surrounding stem cells [97].

BES1 is another transcription factor of the BR signaling pathway and shares 88% sequence identity with its closest paralog, BZR1. BES1 also tightly connects the BR pathway to other hormone responses in *Arabidopsis*. In the gain-of-function *bes1-1D*, a dominant mutation that leads to overaccumulation of BES1, some auxin-responsive genes are induced [99]. The auxin-responsive gene *SAUR15* is upregulated in the *bes1-1D* mutant and induced by BR without increasing the endogenous auxin levels [100]. Interestingly, the auxin efflux carriers PIN4 and PIN7, which maintain the distribution and endogenous auxin gradient, are controlled by BES1 [101]. When grown in the dark, the phenotype of the *bes1-1D* mutant was shown to be similar to *bzr1-1D* [77]. However, both mutants have distinct light-grown phenotypes that are consistent with their effects on the feedback regulation of BR biosynthetic genes [99]. While the *bzr1-1D* mutant has reduced BR levels and lower expression of the BR biosynthetic pathway gene *CONSTITUTIVE PHOTOMORPHISM AND DWARFISM* (*CPD*), *bes1-1D* has only a small effect on *CPD* gene expression [99]. This suggests that BZR1 plays a major role in the activation of the BR negative feedback pathway that inhibits BR biosynthetic genes [77]. Interestingly, another BR biosynthetic gene, *BREVIS RADIX* (*BRX*), is under a feedback loop during *Arabidopsis* root development and mediates feedback between auxin and BR signaling [102]. In the future, it would be interesting to evaluate the effects of BZR1 on *BRX* gene expression in different root tissues at different BR levels.

From the molecular point of view, the question that needs to be addressed is: what is the conversion point of different hormone signals at different stages of development, at different organs and under different hormone levels? Unfortunately, there is no clear answer yet. Studies on the relationship between BR and auxin might clarify the complex biological significance of the question above.

**Figure 3:**
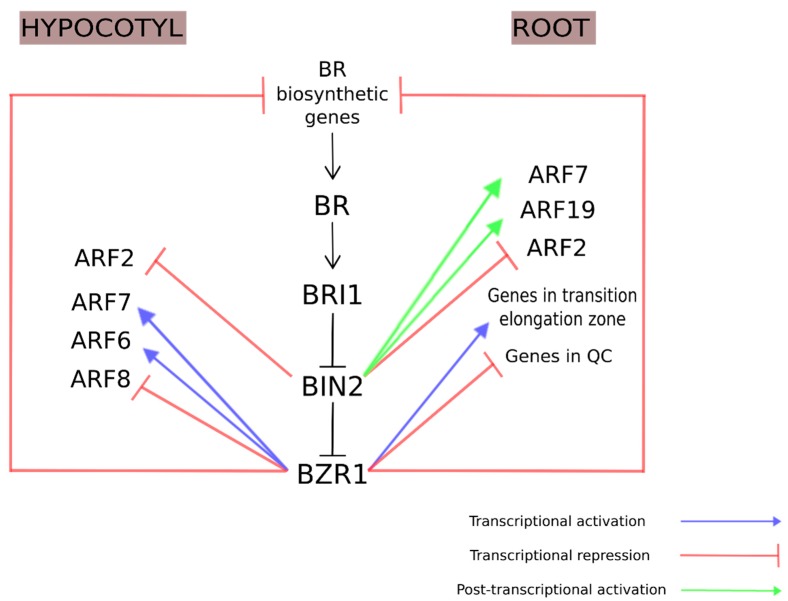
Schematic working model of regulatory interactions between BR and auxin in root and hypocotyl growth. The green arrows represent the post-transcriptional activation of AUXIN RESPONSE FACTOR (ARF) by BRASSINOSTEROID-INSENSITIVE 2 (BIN2). The blue arrows represent the transcriptional activation of ARF and auxin-responsive genes in the root transition elongation zone by BRASSINAZOLE RESISTANT 1 (BZR1). The red arrows represent the transcriptional repression of ARF and auxin-responsive genes in the root quiescence center (QC) by BZR1. The negative feedback of biosynthetic genes coordinated by BZR1 in both root and hypocotyl elongation is also represented by red arrows.

In summary, Figure 3 shows a schematic working model for the cross-talk between BR and auxin. The concept behind this model is a mechanism involving the control of BR–auxin interaction by a tissue-specific transcriptional/post-transcriptional regulation circuit in a hormone dose-dependent manner. A detailed molecular link between the interaction of BR and auxin in plant growth remains elusive, and further investigations will be essential to understand the spatiotemporal pattern of BR–auxin cross-talk.

### 3.2. Brassinosteroids and Gibberellins

#### 3.2.1. BR–GA Cross-talk: The Signaling Model

A long-standing theme in plant development is how, when and where hormonal cross-talk orchestrates a myriad of developmental cues while simultaneously transmitting environmental inputs. Over the years, this multidynamic mapping of hormonal signaling has elegantly been deciphered by transcriptional and post-transcriptional regulatory mechanism models. Therefore, it is not surprising that there has been a strong effort over the last two decades, particularly in the last six years, to develop an improved integrated model of BR–GA coordination. To date, three out of eight classes of hormones in plants have been identified as major classes of growth-promoting hormones which include auxins, gibberellins, and brassinosteroids. Despite their interdependences in playing a wide range of growth and developmental processes in different contexts throughout the life cycle of plants, they also act through a woven network, regulating themselves and several downstream effects [103].

**Figure 4:**
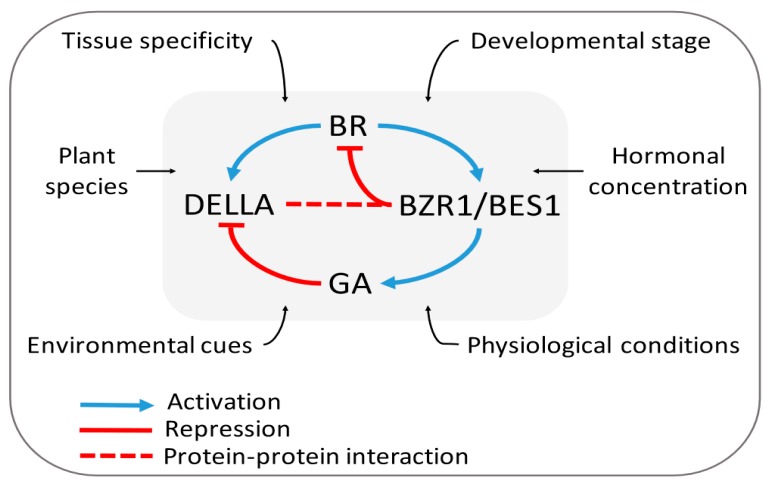
The integrated model for BR–Gibberellin (GA) cross-talk. BR activates BRASSINAZOLE RESISTANT 1/BRI1-EMS SUPPRESSOR 1 (BZR1/BES1) to promote GA biosynthesis and production. As a consequence, GAs degrade DELLA proteins, releasing their repressive action on BZR1/BES1 activity. Some critical factors, emphasized above, may influence and alter this interaction over time and should be considered when discussing BR–GA coordination in plants (e.g.; BR-induced accumulation of DELLA at dawn in the early stages of *Arabidopsis* development).

Gibberellins are a group of tetracyclic diterpenoids, synthesized by a multistep process, which act as mobile signals [104] with diverse intermediates being processed into different cellular compartments [105]. Several studies have shown the complex spatiotemporal regulation of their biosynthesis in different tissues, cell types and developmental phases [106]. GAs’ distribution and mobility, recently clarified through the report of two GA transporters (i.e.; the nitrate transporter 1/peptide transporter family (NPF) [107] and SWEET13/14 proteins [108]) have long been described to long-distance movement, but their combinatorial effects on GA activity at a cellular resolution have only recently been clarified through novel approaches using the GA biosensor (termed GPS1) [109] and a fluorescently labeled version of active GA_3_ and GA_4_ (termed GA–FI) [110]. In contrast to this multifaceted regulation, their signal transduction mechanism seems to be relatively straightforward, whereas GA-induced DELLA degradation acts as a central regulatory switch for GA signaling (Figure 4). Briefly, active GAs are recognized and bound to their receptor GIBBERELLIN INSENSITIVE DWARF1 (GID1), which, in turn, binds to the N-terminal of DELLA proteins, relieving their repression by promoting their degradation via the ubiquitin–proteasome pathway [111]. Of note, the existence of a DELLA-independent signaling pathway has also been reported through the increase of [Ca^2+^]_cyt_ within a few minutes after GA treatment [112].

The most convincing evidence of this tangled interaction between BR and GA came in the late 1990s, with the discovery of the remarkably resembled phenotypes (being de-etiolated in the dark and dwarf stature in the light) between GA- and BR-deficient *Arabidopsis* mutants [96,113,114,115,116,117]. Subsequently, several detailed physiological, metabolic and genetic studies in pea (*Pisum sativum*) [118], mung bean (*Vigna radiate*) [35], cucumber (*Cucumis sativus*) [119], rice (*Oryza sativa* L.) [120], and, in particular, in *Arabidopsis* [121], started to reveal evidence of a cooperative and interdependent relationship between BRs and GAs, but with multiple layers of this complex interaction acting in a species-, tissue-, and dose-dependent manner. The elusive nature of such responses in this complex interplay was clarified further when, in 2012, a direct physical cross-talk between their signaling pathways was revealed, and the signaling model was proposed (Figure 4). In fact, DELLA not only interacts with BZR1/BES1, but also exerts an inhibitory effect on BZR1 transcriptional activity [122,123,124]. This mechanistic molecular framework became the stepping stone towards expand the understanding of the integration between BR–GA activities, whereas if DELLAs inhibit BZR1 activity and GA-induce DELLA degradation, GA and BR should affect the expression of BZR1 target genes similarly in the control of plant growth and development.

Consequently, this strengthened notion was further examined and validated through the coregulation of common target genes mediated by the BZR1–DELLA interaction. Bai and coworkers elegantly firstly demonstrated that 419 (35%) out of 1,194 genes differentially expressed in *ga1-3* (GA-biosynthesis deficient) compared to WT plants, were also affected in the *bri1-116* (BR-insensitive) mutant, of which 387 (92.3%) of the coregulated genes were affected in the same way by these mutants. Secondly, they analyzed RNA-sequencing data from GA-treated WT and GA-treated WT grown on PPZ (a specific inhibitor of BR biosynthesis) medium, identifying 3,570 and 1,629 differentially regulated genes, respectively. Again, this striking data suggested that around 66.7% of GA-regulated genes require BR, emphasizing the important role of BR in the GA regulation of genome expression [122]. Consistent with these data, other groups showed that hypocotyl elongation promoted by GA was eliminated in *Arabidopsis* seedlings with reduced BR biosynthesis (i.e.; *de-etiolated-2* (*det2*) mutants or brassinazole (BRZ) treatment), indicating that cell elongation largely relies on the appropriate action of both hormones [123,125]. Later experiments, discussed in more detail below, showed that the capacity of GA to rescue the growth defects of BR mutants is dependent on the developmental stage, on the physiological conditions and also on the fact that the GA pathway is only one of the branched pathways of BR-regulated growth [126].

Even in the absence of BR, GAs might also regulate BZR1-dependent gene expression, at least in part, since GA treatment slightly increases the dephosphorylation state of BZR1, its active form, likely through phosphatase PP2A proteins [124]. This action might explain the increased BZR1–DNA binding in vivo and GA-induced the modulation of BR transcriptional outputs [122]. Interestingly, this slight rise in the dephosphorylated BZR1 concentration was abolished in the presence of the protein phosphatase inhibitor okadaic acid (OA), and, in the same manner, in paclobutrazol-treated plants, which also showed a reduced level of two PP2AB’ subunits (PP2AB’α and PP2AB’β) [124]. In future studies, it will be exciting to elucidate how GA and DELLA act on PP2A regulation to promote the phosphorylation state of BZR1. The fact that DELLA proteins interact exclusively with the dephosphorylated BZR1 indicates that BR signaling enhances GA signaling by promoting the BZR1–DELLA interaction and, therefore, the alleviation of DELLA’s restraint imposed on GA-mediated growth [124]. This BZR1 titration might explain why, surprisingly, BR was shown to strongly increase the abundance of the DELLA protein at the early elongation stages postgermination in *Arabidopsis* [125]. However, on the other hand, another group showed that neither BR treatment, nor BR biosynthesis or signaling mutants affected the accumulation of DELLA proteins in seedlings of 12-day-old *Arabidopsis* plants [124]. One explanation for these seemingly contradictory findings might be related to the developmental stage and tissue studied, evidencing the complexity of this hormonal interaction.

#### 3.2.2. The Expanded and Integrated Model

Although this attractive signaling model could shed some light on the BR–GA interaction, recent detailed results on the potential interaction between BR and GA biosynthesis brought an informative readout at the level of hormonal biosynthesis, providing a novel expanded and integrated model of BR–GA cross-talk. Nonetheless, it is worth mentioning that a previous study had already demonstrated that BR promotes the expression of GA biosynthetic genes, and that DELLA can also modulate negative feedback in the BR biosynthetic genes by preventing the DNA-binding ability of BES1 and BZR1 proteins [125]. This overlooked biosynthetic cross-talk gained some attention following the recent demonstration by independent groups that the active GA contents (and various GA intermediates therein for *Arabidopsis*) were reduced in *Arabidopsis* (*ASKθ-oe*) and rice (*d11*, *GSK2oe*, and *dlt*) BR deficient mutants in comparison to those in WT plants. Similarly, an increase in the GA_1_ level in BR-accumulating rice (*Do* and *m107*) lines was observed [126,127]. Strengthening these findings, and also in line with previous results, the expression levels of two genes (*GA20ox* and *GA3ox*) encoding key enzymes in the rate-limiting step of GA production were shown to be impaired in BR mutants, but were also strongly increased after BR treatment in *Arabidopsis* and rice plants, clearly indicating that BR influences GA biosynthesis in dicot and monocot plants. Such findings became more evident through the use of bioinformatics, ChIP, and in vitro DNA binding studies, which demonstrated that BZR1/BES1 can directly bind to the target promoters of *GA20ox*, *GA3ox*, and *GA2ox* from *Arabidopsis* and rice plants. These analyses revealed that BZR1/BES1 binding *cis*-elements are highly enriched on these promoters, including the BR-response element (BRRE, CGTG^T^/_C_G), G-box (CACGTG) and a type of E-box (CATGTG) in rice, and a non-E-box (AA^T^/_A_CAAnnnC^C^/_T_T) motif in *Arabidopsis* [126,127]. Importantly, there was a higher enrichment of BES1 on these promoters followed by BR treatment, evidencing that the dephosphorylation of BZR1/BES1 increases GA production.

Extending the analysis to the effects of *GA20ox* expression on BR mutant phenotypes, complementation of the *bri1-301* mutant with *GA20ox1* under the control of the *BRI1* promoter restored various growth defects of the BR-deficient seedlings, demonstrating that some defects are related to GA deficiency [126]. Additionally, in contrast with the previous observations that BR- deficient and -insensitive mutants conferred insensitivity to GA, two independent groups demonstrated that externally applied GA could restore growth defects of *Arabidopsis* and rice BR mutants [126,127]. However, the developmental stage, environmental context, tissue specificities, hormone concentration, and species must be considered during the study of this positive loop between GA and BR.

At this stage, the proposed model postulates that BR activates BZR1/BES1 post-translationally to induce GA biosynthesis, and the increased GA induces DELLA degradation to further release BZR1/BES1 activity (Figure 4). Although this expanded model has incited a debate around the relative importance of the biosynthesis and signaling pathways [128,129,130], it is essential to highlight the applicability of this model to different contexts, as described above. Nevertheless, recent mathematical modeling and analysis of BR–GA cross-talk revealed that the signaling model (BZR1/BES1–DELLA interaction) exerts a stronger influence on the dynamics of the BR and GA signaling pathways than the BZR1/BES1-mediated biosynthesis of GA. Besides, the stability of this feed-forward model is mainly dependent on the mechanisms involved in the phosphorylation state of BZR1/BES1 proteins and the cellular localization of these processes [131].

#### 3.2.3. Is the BR–GA Antagonism an Alternate Strategy to Tackle Biotic and Abiotic Stresses?

As pointed out above (Figure 4), the high degree of complexity in BR–GA cross-talk can be attributed to several factors that profoundly influence BR and GA homeostasis. One interesting example is the fact that plant pathogens may exploit endogenous hormones or produce deceptive BR or GA (or mimics thereof) signals for their own advantages in order to manipulate and subdue their host’s immunity. In rice, the root pathogen *Pythium graminicola* uses endogenous BR as a virulence factor and manipulates the host BR signaling to alleviate effective GA-mediated defenses. In this case, BR suppresses GA biosynthesis and induced GA repressor genes (*GA2ox3*) that indirectly stabilize the rice DELLA protein, SLENDER RICE 1 (SLR1) [132]. Thus, as a virulence strategy, BR promotes susceptibility to this particular pathogen, disarming the plant’s defense signaling circuitry, which is in contrast to the protective effects of BRs that have been unveiled so far against myriad fungal, viral, and bacterial pathogens.

Intriguingly, the same BR–GA antagonism mechanism was reported in the submergence response in rice [133]. The tolerant *M202-Sub1* line adopts a quiescent strategy that limits shoot elongation during transient flooding, conserving energy until floodwaters retreat. The increased BR level in these plants during submergence induces a GA catabolic gene (*GA2ox7*) and the DELLA protein SLR1, restricting growth through the repression of GA signaling. In keeping with this data, BR pretreatment of the intolerant *M202* line before inundation was shown to restrict shoot elongation, conferring submergence tolerance [133].

In contrast to the antagonistic control of BR on GA metabolism, the positive effect of BR on DELLA protein stability may offer a mechanistic explanation for the abiotic stress tolerance conferred by BR. The positive correlation between DELLA protein levels and tolerance to abiotic stresses has been attributed to elevated expression of reactive oxygen species (ROS)-scavenging enzymes [134]. However, the dynamics and stability of DELLA and BZR1 protein complexes in response to pathogen and abiotic stresses remain elusive.

In summary, the intricate interconnection of BR with GA illustrates the functional versatility of these hormones whereby the integration of their outputs and signals of adverse conditions stimulates a balance between plant defense and growth responses. Nonetheless, the understanding of how the BR–GA interplay acts in biotic and abiotic stresses is still far behind that of the classic defensive hormones JA, ET, and SA.

### 3.3. Brassinosteroids and Cytokinins

Cytokinins are a group of phytohormones that play important roles in several biological processes, such as the development of aerial and subterranean organs, light responses, mineral enrichment, and responses to abiotic stresses [135,136,137]. The key enzymes involved in CK metabolism are isopentenyltransferases (IPTs), which are responsible for the biosynthesis of bioactive CKs, and CK oxidases/dehydrogenases (CKXs), which are responsible for the inactivation of bioactive CKs [135], both targets of BR-mediated responses.

The main interplay between CKs and BRs seems to be related to plant growth regulation [138]. The *CKX3* gene from *Arabidopsis* directs the breakdown of CKs, and when overexpressed under the control of a root-specific promoter *PYK10*, lower CKs levels in roots were observed, causing a reduction of root growth and also a weak reduction of leaf growth in *Arabidopsis* [136]. On the other hand, plants ectopically expressing both *CKX3* and *BRI1* present a synergistic increase in leaf and root growth. In agreement, *PYK10::CXK3* transgenic plants treated with exogenous BR showed an accentuated growth of lateral roots compared to WT plants, strongly suggesting a cross-talk between BRs and CKs that controls growth and developmental processes [138].

Moreover, the interplay between BR and CK can be observed in CK-induced anthocyanin production [139]. *Arabidopsis* mutant seedlings defective in BR biosynthesis (*dwf4*, *dwf4-102*, and *psc1*) and BR signaling (*bri1-4*), were submitted to different trials to evaluate the effects of BR on CK-induced anthocyanin accumulation. The *dwf4* and *bri1-4* plants presented reduced CK-induced accumulation of anthocyanin, but when WT plants were treated with exogenous BR, an increase in anthocyanin levels was observed. Similarly, CK-induced expression of anthocyanin biosynthetic genes, such as *dihydroflavonol reductase*, *leucoanthocyanidin dioxygenase*, and *UDP-glucose:flavonoid-3-O-glucosyl transferase*, presented an accentuated reduction in the *dwf4-102* and *bri1-4* lines compared to WT. In addition, WT plants treated with CK presented higher expression of transcription factors related to anthocyanin production, including anthocyanin *pigment 1* (*PAP1*), *glabra 3* (*GL3*), and *enhancer of glabra 3* (*EGL3*), but the same was not observed in the *bri1-4* and *dwf4-102* lines. These data provide evidence that BR may boost CK-induced anthocyanin biosynthesis by positively mediating the expression of biosynthesis and signaling genes as well as transcription factors involved in both cases [139].

As with various phytohormones, later evidence suggested that CKs play important roles in several abiotic stress responses [140,141,142]. Studies of the gain- and loss-of-function of selected genes suggested that CKs negatively regulate several stress responses. Constitutive overexpression of *CKX* genes was implicated in CK deficiency and an increase in drought and salt tolerance, while the loss-of-function of *IPT* genes also led to increased stress tolerance due to decreasing bioactive CK levels [137]. Parallel experiments showed that the negative relation between the CK content and stress tolerance might be associated with a mutual interplay between CKs and ABA [143]. The treatment of *CKX* overexpressing lines and *IPT* silencing lines with exogenous ABA similarly resulted in the decrease of biologically active CK contents. Nevertheless, CK-deficient mutants were shown to be more sensitive to ABA compared to WT plants, leading to a higher induction of ABA-signaling marker genes under stress conditions (e.g.; *AIL1*, *COR47*, *RAB18*, *RD29B*, and *SAG29*) and subsequently, enhancing stress tolerance. These data suggest that the elevated stress tolerance in CK-deficient plants compared to WT plants may be related to the ability of these mutant plants to react more quickly to ABA and stressful conditions by further repression of the CK signaling pathway.

Besides the interplay of ABA and CK in stress tolerance regulation, other studies in rice (*Oryza sativa*) showed that BR might be associated with CK-mediated responses to drought stress in a different way. Rice transgenic lines expressing the *IPT* gene driven by a stress- and maturation-induced promoter (*P_SARK_*) presented an increase in CK content before the beginning of senescence as well as the upregulation of several genes involved in the activation of BR signaling (*BRL3*, *BRI1*, *BH1*, *BIM1*, and *SERK1*) and biosynthesis (*DWF5* and *HYD1*), in water-stressed and well-watered plants. Under stress conditions, this resulted in a delay in stress symptoms such as leaf rolling, senescence, and decreased photosynthesis activity, which contributed to an increased grain yield [144].

It is well documented that CKs have an important role in the source/sink relationship [145]. During the vegetative and premature reproductive stages of cereal plants, the assimilated carbon is temporarily stocked in the stem and leaf sheaths in carbohydrate form. In the later stages of plant development, these stored compounds are subsequently remobilized to reproductive sink tissue as flowers and grain filling [146]. However, the maintenance of source/sink homeostasis is a major challenge during stress conditions, causing yield losses. In *P_SAPK_::IPT* lines, the increase of CK content enabled the maintenance of source strength during drought stress, keeping higher yields compared to WT plants. It is also known that the application of BR is a powerful biotechnological tool to enhance crop yield [147,148,149,150,151,152]. According to the presented scenario, the changes in hormonal profile, including the upregulation of BR-related genes, can modify the source/sink relationship, providing a strong sink capacity to *P_SAPK_::IPT* line plants during water stress. Together, these data suggest that BR–CK cross-talk may contribute to the modification of source/sink relations, improving crop yield and stress responses.

**Figure 5:**
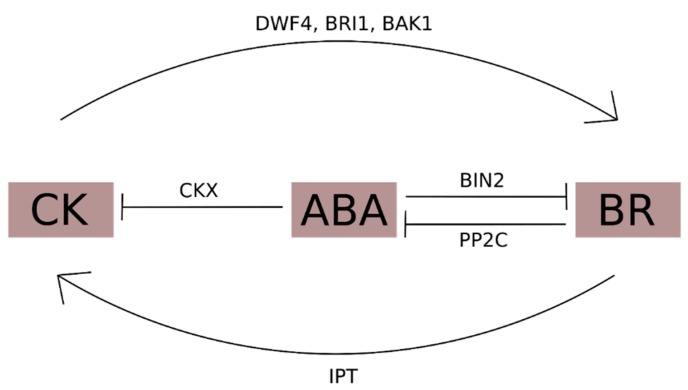
A putative interplay between brassinosteroid (BR), cytokinin (CK), and abscisic acid (ABA). ABA is responsible for inhibiting BR effects during stress conditions by upregulating *BIN2* (BRASSINOSTEROID-INSENSITIVE 2), a major negative regulator of BR signaling, whereas BR is responsible for inhibiting ABA effects during growth processes through *PP2C* (PROTEIN PHOSPHATASE 2C), a major negative regulator of Snark proteins (positive regulators of ABA signaling). ABA is also responsible for inhibiting CK signaling by upregulating *CKX* (CK oxidases/dehydrogenases), which play a major role in inactivating bioactive CKs. Despite ABA’s role, BR and CK present positive interactions. While CK upregulates BR biosynthetic (*DFW4*) and signaling (*BRI1*, *BAK1*) genes, BR upregulates IPT (isopentenyltransferases), which are major enzymes responsible for the biosynthesis of bioactive CKs.

It has been observed that BR and ABA present antagonistic actions [153]. BR-mediated signaling is regulated by ABA through the upregulation of *BIN2* and downregulation of genes from the PP2C family, causing decreased activity of the BR signaling pathway [153]. The relative expression of three members of the *PP2C* family (*PP2C7*, *PP2C6*, and *PP2C53*) was increased in WT plants under water stress. However, the expression of *BIN2* was upregulated in plants of *P_SAPK_::IPT* lines [153]. ABA is responsible for inhibiting BR effects during stress conditions. Therefore, the observed hormonal profile in the mentioned study and its consequences may be due to the interplay not only between CK and BR, but also between the three hormones—CK, ABA, and BR—in a complex manner that remains unclear [144]. The role of ABA in abiotic stress and its cross-talk with BR are discussed in more detail in Section 3.5. A suggested interplay between BR, CK, and ABA is represented in Figure 5.

### 3.4. Brassinosteroids and Ethylene

Ethylene is a gaseous phytohormone with a simple structure. Because volatile substances move rapidly, they can act as regulators and coordinators of several growth and development processes, both in the tissue and in the whole organism, as well as facilitating plant-to-plant communication. Although the main function attributed to ethylene is fruit ripening promotion, other physiological processes, such as seed germination, senescence, and responses to abiotic and biotic stress factors, are also regulated by this hormone [154]. Ethylene biosynthesis requires the participation of five major components: the amino acid methionine which is converted into S-adenosyl methionine (SAM²) and subsequently modified by the ACC-synthase enzyme (ACS) to form 1-aminocyclopropane-1-carboxylic acid (ACC), the direct precursor of ethylene. In turn, ACC is converted by the enzyme ACC-oxidase (ACO) into ethylene, a stable compound that can be transported throughout the plant [155].

Brassinosteroids influence ethylene biosynthesis mainly by regulating *ACS* and *ACO* activities [156]. The cross-talk between these two phytohormones presents two scenarios, with BR regulating ethylene production at the transcriptional and post-transcriptional levels. Regarding protein regulation, previous studies in *Arabidopsis* indicated that seedlings treated with exogenous BR show elevated levels of ethylene biosynthesis, at least partly through an increase in ACS5 protein stability by elevating its half-life [156]. Additionally, other studies have already found that BR may also regulate ethylene biosynthesis through the induction of *ACS5* gene expression in *Arabidopsis* [157].

**Figure 6:**
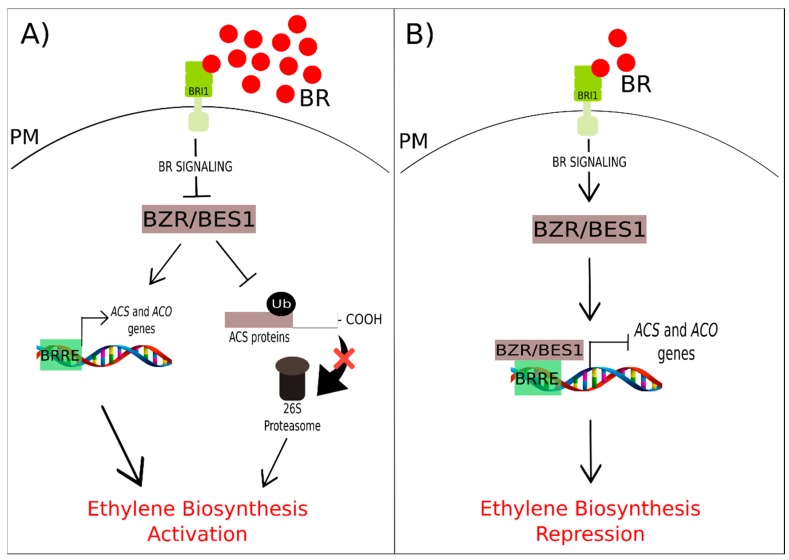
A general simplified model of BR and ethylene cross-talk. The perception of BR begins in its receptor BRASSINOSTEROID INSENSITIVE 1 (BRI1) which activates BR signaling, which, in turn, controls ethylene biosynthesis in a dose-dependent manner. (**A**) High levels of BR decrease the activity of BRASSINAZOLE RESISTANT 1/BRI1-EMS SUPPRESSOR 1 (BZR1/BES1), the major transcription factors of the BR signaling pathway while enhancing the stability of the 1-aminocyclopropane-1-carboxylic acid (ACC)-synthase enzyme (ACS) proteins by preventing its degradation by the 26S proteasome and consequently, activating ethylene biosynthesis. (**B**) Low levels of BR increase the activity of BZR1/BES1, which, in turn, bind to the promoter of the *ACC-synthase* (*ACS*) and *ACC-oxidase* (*ACO*) genes, inhibiting their transcription and consequently, repressing ethylene biosynthesis. PM represents the plasma membrane.

The regulation of ethylene biosynthesis by BR happens in a dose-dependent manner, where BRs can be positive as well as negative regulators, depending on the exogenous application dose (Figure 6) [158]. High levels of BRs stimulate ethylene biosynthesis by enhancing the stability of the ACS protein by preventing its degradation by the 26S proteasome. On the other hand, low levels of BRs repress ethylene biosynthesis by increasing the activity of *BZR1/BES1*, the two major BR signaling pathway transcription factors that inhibit the transcription of *ACS* genes [158]. Experiments with banana fruit (*Musa acuminata* L.) showed that BZR proteins bind specifically to BRRE elements (CGTGT/CG) of at least one *ACS* gene (*MaACS1*) and two *ACO* genes (*MaACO13* and *MaACO14*) in this species. An expression analysis showed that the expression of *MaBZR1*, *MaBZR2*, and *MaBZR3* decreases continuously during fruit ripening. Moreover, *MaBZR1* and *MaBZR2* are capable of suppressing the transcription of these three ethylene biosynthetic genes, which is increased during the fruit ripening process. Additionally, the exogenous application of BR promotes banana fruit ripening due to the acceleration of *MaACS1*, *MaACO13*, and *MaACO14* expression, and consequently, ethylene production occurs, confirming the action of BZR proteins as transcriptional repressors of ethylene biosynthesis [159].

The application of exogenous BR can also accelerate postharvest ripening, enhancing the development of quality attributes and consequently, promoting ethylene production in *Solanum lycopersicum* by increasing transcriptional levels of *ACS2* and *ACS4* genes [160]. Tomato fruits with enhanced BR levels or BR signaling due to overexpression of the BR biosynthetic gene *DWARF* and the signaling gene *BRI1* showed elevated ethylene production and quick-ripening, respectively [161,162]. On the other hand, tomato plants silenced for the *BRI1* gene and insensitive to BR presented no changes in ethylene accumulation, ACC content, and ACS and ACO activities during BR treatment, reinforcing that BRI1 downstream components may be involved in ethylene accumulation [163], as also suggested by Lv et al. 2018 [158].

Ethylene is also known to be involved in a range of stress responses, such as heat stress [164] and pathogen and pest attacks [165]. Studies using mutants that are deficient and insensitive to ET showed higher thermal and salt tolerance when 24-epibrassinolide (EBR) was applied. EBR was capable of increasing the survival rates of the ET-insensitive mutant *ein2* under heat stress in *Arabidopsis* plants. Moreover, the treatment of *Brassica napus* seeds with EBR reduced the inhibition *ein2* mutant germination under salt stress, reverting this line’s hypersensitivity to salt to a level similar to those of WT plants [166].

Lettuce plants present high emission of ET and an increase in ACC content during salt stress [167]. However, the increase in ethylene production under salt stress leads to the inhibition of plant growth and induction of senescence and consequently, premature death [168]. The treatment of lettuce plants under salt stress with DI-31, a brassinosteroid analog, was shown to be capable of reducing the ACC content and consequently, ET production, avoiding premature death, alleviating weight loss, and showing a good protective effect of BR against salinity.

### 3.5. Brassinosteroids and Abscisic Acid

Abscisic acid (ABA) is a phytohormone that is involved in a wide range of plant responses and is essential for plant development and survival. The hormone acts as a major abiotic stress sensor, leading to protective responses such as stomatal closure, seed dormancy, and inhibition of growth and germination [169,170,171,172,173]. Even in the early stages of plant development, ABA drives stress tolerance and/or avoidance mechanisms, helping plants to survive in adverse conditions [174].

The serine-threonine kinases SnRK2.2/2.3/2.6 (SNF1-related protein kinases) play a central role in the ABA pathway response as positive regulators of ABA signaling [173,175,176,177]. The kinases regulate the expression of stress-responsive genes and transcription factors, leading to ABA-related responses. The kinases’ activity is modulated by their interactions with PHOSPHATASE 2C (PP2C), which inactivates SnRK2s by dephosphorylation [178]. In the presence of the hormone, the complex formed by ABA and PYL/PYR/RCAR receptors inactivates the phosphatase by blocking the substrate’s entry [179,180,181,182,183].

Despite the essential roles of PP2C and SnRK2s in activating ABA responses, their effects in plant cells are influenced by cross-talk with other phytohormones. For example, seed dormancy is affected by the interplay of abscisic acid with gibberellins and ethylene [184]. Also, stomatal movement is regulated under stress by jasmonic acid, cytokinins, ethylene, auxin, and also, brassinosteroids [185,186]. In general, under favorable conditions, the cross-talk between growth-related hormones and ABA results in the attenuation of ABA-related responses by diverse molecular mechanisms, allowing plant growth and development.

The antagonism between ABA and the growth-related hormone brassinosteroid has been known for several years. The negative cross-talk between these hormones has been observed during seed germination, early seedling development, root growth, and stomatal closure [153,187]. Moreover, mutants with defective BR signaling (i.e.; *bin2-1*, *bri1*, *constitutive photomorphogenesis and dwarfism* (*cpd*), and *de-etiolated-2 mutant* (*det2*)) have enhanced ABA sensibility during seed germination, early seedling development, and/or primary root formation [96,187,188,189]. Despite all these observations, the molecular mechanism behind the negative cross-talk remained poorly understood until recently.

Essentially, ABA and BR antagonism includes two types of regulation: post-translational modification at the protein level and transcriptional repression at the gene level. Regarding protein–protein regulation, phosphorylation and dephosphorylation events play a key role in ABA–BR cross-talk. Similar to the ABA signaling pathway, the activity of kinases and phosphatases is crucial to brassinosteroid sensing and responses. The presence of brassinosteroid triggers the activation of the BRI1 kinase-like receptor, the kinases BAK1 and BRI1 and the phosphatase BSU1. This phosphatase is responsible for the dephosphorylation of the kinase BIN2, a major repressor of BR signaling [190].

A considerable body of evidence indicates that BIN2 is one of the key players in ABA–BR cross-talk. This kinase can interact and phosphorylate *Arabidopsis* SnRK2.2 and SnRK2.3 in vitro [191]. BIN2-mediated phosphorylation was shown to increase SnRK2.3 activity in vitro. While the in vivo overexpression of SnRK2.3 caused ABA hypersensitivity, plants overexpressing SnRK2.3^T180A^ presented sensibility to ABA at levels similar to WT plants. These data suggest a role of T180 phosphorylation in ABA signaling in vivo. 

BIN2 activity also affects another ABA pathway element downstream of SnRK2s, the basic leucine-zipper (bZIP) transcription factor ABA Insensitive 5 (ABI5). In the presence of ABA, ABI5 regulates seed germination, and seedling growth, leading to seed dormancy and growth arrest responses [192,193,194]. Moreover, ABI5 activates *LATE EMBRYOGENESIS ABUNDANT* (*LEA*) genes in vegetative tissues [194]. A recent study showed that ABI5 interacts with BIN2, which then phosphorylates ABI5 in vitro [195]. In vivo, seeds from the gain-of-function *bin2* mutant (*bin2-1*) presented higher expression of ABI5 target genes during ABA-treatment compared to the triple knockout mutant (*bin2-3 bil1 bil2).* The effect of BIN2 on ABI5 phosphorylation and target regulons expression indicates that BIN2 might modulate ABA signaling during seed germination and early seed development.

Despite all evidence showing some ABA pathway key elements are targets of BIN2, a recent study suggests that ABI1 and ABI2 [166] might regulate kinase activity [196]. Overexpression of the PP2C family phosphatases ABI1 and ABI2 in *Arabidopsis* resulted in decreased expression of the gene markers of BR suppression: CPD and DWF4. Moreover, phosphatase overexpression led to the accumulation of BES1 in its dephosphorylated form. Similar results were previously observed in *abi1* and *abi2* mutants after ABA treatment [153]. The direct interactions between ABI1, ABI2, and BIN2 could be the mechanism behind these effects: the BR-repressor BIN2 is dephosphorylated by the phosphatases, leading to the accumulation of active BES1 [196]. This mechanism may also explain why only BIN2 extracted from ABA-treated seedlings can phosphorylate ABI5 in vitro [195].

Aside from BIN2, the kinase BAK1 also seems to be involved in ABA–BR cross-talk. A recent study showed that BAK1 can interact with and phosphorylate SnRK2.6 in vitro [197]. As the kinase SnRK2.6 is the primary regulator of stomatal closure [169,170,171], the lack of BAK1-mediated activation of SnRK2.6 could explain the increased water loss by transpiration observed in *bak1-3* mutants, even during ABA treatment [197].

In addition to protein interactions and post-translational modification, ABA–BR cross-talk also comprises mechanisms of regulation at the transcriptional level. Real-time quantitative reverse transcription-PCR (qRT-PCR) analysis revealed low expression of the ABA-related transcription factors—ABFs, *ABI3*, and *ABI5*—in the gain-of-function mutant *bes1-D* seedlings [198]. On the other hand, *bes1* knockout mutant (*bes1^ko^*) seedlings displayed high expression of the same transcription factors. Additionally, this mutant presented an enhanced ABA response during root growth and seed germination compared to WT plants. The negative role of BES1 in the ABA signaling pathway relies on the interaction of BES1 with TOPLESS (TPL)/HISTONE DEACETYLASE 19 (HDAC19). Once bound to the *ABI3* promoter, BES1 represses *ABI3* expression through histone deacetylation by assembling the TPL–HDAC19 complex. As BES1 cannot interact with the *ABI5* promoter, the decreased expression of this ABA transcription factor observed in *bes1-D* is a consequence of the repression of the upstream element *ABI3* [192,198].

The direct inhibition of *ABI5* expression seems to be controlled by the BZR1 transcription factor. The BR-induced transcription factor binds to G-box sequences present in the *ABI5* promoter, reducing their expression [199]. The regulation of *ABI5* by BZR1 could be the cause of the *ABI5* downregulation in the gain-of-function *brz1-1D* mutants after ABA treatment. Therefore, the ABA insensibility of *bzr1-1D* mutants in root growth assays might be a consequence of ABI5 repression by BZR1, and this could be suppressed by ABI5 overexpression.

**Figure 7:**
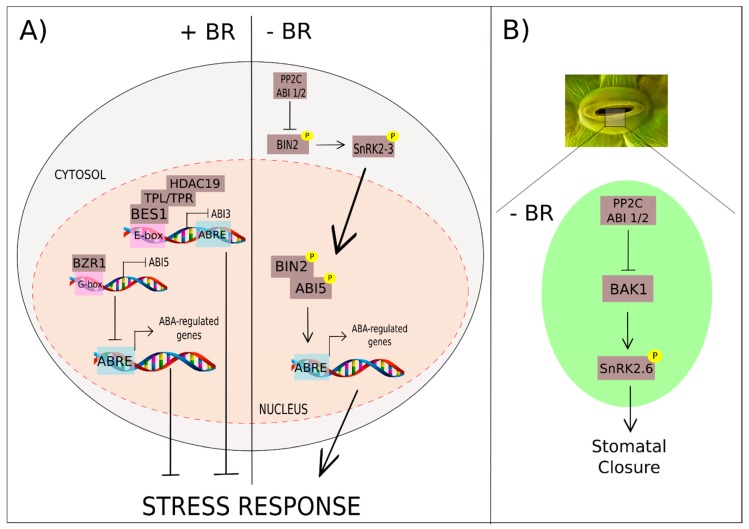
BR and ABA cross-talk relies on protein activity modulation and gene expression regulation. (**A**) In the presence of BR, the complex formed by BRI1-EMS SUPPRESSOR 1 (BES1), TOPLESS/(TPL/TPR) and HISTONE DEACETYLASE 19 (HDAC19) inhibits *ABA Insensitive 3* (*ABI3*) expression by interacting with E-box promoter sequences. The transcription factor BRASSINAZOLE RESISTANT 1 (BZR1) interacts with the G-box sequences of the *ABI5* promoter, leading to gene repression. Repression of the *ABI3* and *ABI5* genes results in lower expression of ABA-regulated genes and decreased stress responses. At low levels of BR, stress responses are stimulated by SnRK2.3 activation by BRASSINOSTEROID-INSENSITIVE 2 (BIN2). Additionally, the BR-repressor BIN2 phosphorylates the transcription factor *ABI5*, resulting in the expression of ABA-related genes. (**B**) In guard cells, BRI1-ASSOCIATED RECEPTOR KINASE 1 (BAK1) phosphorylates the kinase SnRK2.6 at low levels of BR, driving stomatal closure responses. At low levels of ABA, the PHOSPHATASE 2C (PP2C) phosphatase ABI1 and ABI2 repress SnRK2.6 phosphorylation by BAK1 and also the phosphorylation of SnRK2.3 by BIN2, decreasing stress responses related to ABA–BR cross-talk.

Recent findings suggest that ABA–BR cross-talk involves multiple players acting on two fronts: modulation of protein activity and regulation of gene expression. In summary, under optimal conditions, brassinosteroids trigger BR cascade activation and antagonize ABA responses by decreasing *ABI3* and *ABI5* expression during seed germination and seed growth (Figure 7A) [198,199]. The absence of ABA triggers BR responses by repressing BIN2 through PP2C ABI1 and ABI2 phosphatases [196]. However, during BR signaling repression, BIN2 stimulates and enhances ABA responses through direct phosphorylation of SnRK2.3 and ABI5, leading to ABA-modulated seed dormancy and seedling growth arrest responses [191,195]. Under the same conditions, BAK1 increases stomata responses by SnRK2.6 phosphorylation (Figure 7B) [197].

Despite the substantial evidence supporting the molecular mechanism behind ABA–BR cross-talk, key points remain to be clarified. The previously reported lower auto-activation of SnRK2.2 and SnRK2.3 compared to SnRK2.6 suggests the need for activation by an upstream kinase [200]. However, ABA-related SnRK2s have been under investigation for several years, and different studies have shown that kinase auto-activation is sufficient for kinase activity and activation of downstream ABA-related targets [175,200,201,202]. In this sense, further studies are required to elucidate the importance of the brassinosteroid kinases BIN2 and BAK1 in SnRK2s activation and their roles in the ABA response in vivo. Additionally, the understanding of the interplay between brassinosteroid and ABA network elements in particular tissues and plant developmental stages, considering protein spatial distribution and expression, represents a challenge for future studies [192].

### 3.6. Brassinosteroids and Jasmonic and Salicylic Acids

Plants present a range of defense mechanisms whose costs represent a tradeoff between growth and immunity [203,204,205,206], in which phytohormones fulfill central roles in protection against biotic stressors agents. Studies have already proven that BR can induce disease resistance in tobacco (*Nicotiana tabacum*) and rice (*Oryza sativa*) [207] in a complex network which involves crucial functions of the receptor BRI1 and its coreceptor BAK1 [203,208,209,210].

Flagellin 22 (flg22) and chitin are both pathogen- and microbe-associated molecules patterns, also named PAMPs and MAMPs, respectively, which are recognized by the cells of innate immune system as alert signals of invaders. Flg22 binds to its receptor flg-sensing 2 (FLS2), which initiates signals to prevent pathogen proliferation [209,210,211]. Curiously, the binding of flg22 to FLS2 generates an association and transphosphorylation with BAK1 as happens in BR-induced BRI1 signaling, activating FLS2. The activated FLS2 then phosphorylates BIK1 (BOTRYTIS-INDUCED KINASE 1), a receptor-like cytoplasmic kinase responsible for associating with a flagellin receptor complex, triggering plant innate immunity and transducing the target response [209,212,213]. The association of BAK1 as a coreceptor of both BR-induced BRI1 signaling and flg22-induced FLS2 signaling suggests a possible tradeoff between BR and FLS2 signaling responses mediated by BAKI1.

**Figure 8:**
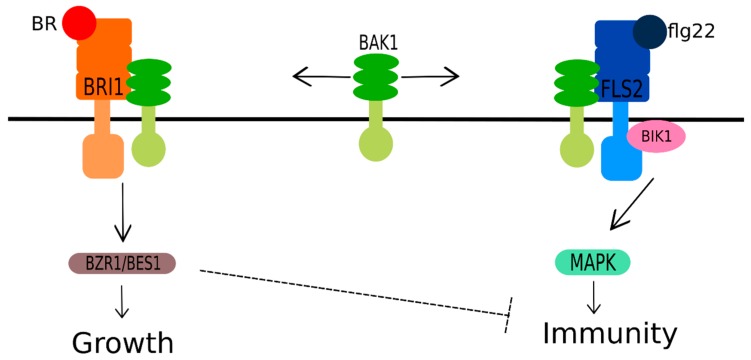
A suggested model of brassinosteroid (BR) regulation of immunity at multiple levels. BAK1 (BRI1-ASSOCIATED RECEPTOR KINASE 1) is considered to mediate the growth and immunity tradeoff because it serves as a coreceptor for both BR-mediated responses via BRI1 and innate immunity mediated responses via flg-sensing 2 (FLS2). The scheme suggests an inhibition of FLS2-mediated immune signaling by BR, independent of complex formation with coreceptor BAK1, when the inhibition occurs downstream of BAK1. BRI1 (BRASSINOSTEROID-INSENSITIVE 1) represents a BR receptor; flg22 (flagellin 22) is a type of pathogen- and microbe-associated molecule pattern (MAMP/PAMP); FLS2 (flg-sensing2) is a flg22 receptor; BIK1 (BOTRYTIS-INDUCED KINASE 1) is a coreceptor of FLS2; BZR1/BES1 (BRASSINAZOLE RESISTANT 1/BRI1-EMS SUPPRESSOR 1, respectively) are the major transcriptional factors of the BR signaling pathway; MAPKs (mitogen activated protein kinases) are a class of marker proteins which indicate various stress conditions.

However, another study suggested the potential existence of BAK-independent immune signaling [214]. *Arabidopsis* plants treated with both exogenous BR and flg22 showed decrease of flg22-induced MAMP-triggered immunity responses (MTI) by BR. However, on the other hand, flg22 did not affect the BR-induced responses. Additionally, when BR and flg22 were applied separately, they induced distinct gene profiles and biological responses (i.e.; the treatment with flg22 induced the stress markers ROS and MAPKs (mitogen activated protein kinases), which were not observed in plants treated only with BR). These data suggest the inhibition of FLS2-mediated immune signaling by BR, independently of a complex formation with its coreceptor BAK1 and associated downstream phosphorylation when different pools of BAK1 exist and are not interchangeable: the BAK1 recruited by FL2S complex is different from BAK1 recruited by BRI1 signaling [214]. Another independent study corroborated these ideas by providing evidence that the association between BRs and MTI responses depends on the endogenous levels of BR and BRI [215]. A possible mechanism to explain the relation of BR to plant innate immunity is represented in Figure 8.

The importance of jasmonic acid (JA) and salicylic acid (SA) for the plant innate immune system is well characterized [216,217]. These hormones generate and transmit distinct defense signals which are capable of influencing each other through a complex network of synergistic and antagonistic interactions [218,219], allowing the plant to efficiently create a quick and precise defense reaction to causal agents of many types of biotic stress. Previous studies have already shown a mutually antagonistic activity of JA and SA in plant innate immunity [220,221,222]. Exogenous application of JA can dramatically decrease the SA content in rice, which suggests that JA can suppress the SA pathway [223]. However, recent studies revealed a diverse and complex interplay between BR, JA, and SA.

A negative role of BR in the defense against brown planthopper (BPH, *Nilaparvata lugens*) was observed in rice (*Oryza sativa*). BPH infestation suppressed the BR pathway, decreasing the expression of signaling genes (*BRI1* and *BZR1*) and the BR concentration, while successively activating SA and JA pathways. Moreover, the application of exogenous BR downregulated the expression of genes related to the SA pathway, such as the biosynthetic genes *ICS1* and *PAL*, and reduced SA content, while it upregulated genes related to the JA pathway, like *MYC2*, *AOS2*, and *LOX1*, and increased the JA content during BPH infestation in WT plants [224]. However, this work also observed that BR-mediated suppression of the SA pathway might be associated with the JA pathway. To further corroborate this fact, JA-deficient mutant *og1* and JA-insensitive mutant *col1-18* were submitted to BR exogenous application. The transcription levels of *ICS1* and *PAL*, two SA biosynthetic genes, were not suppressed and SA levels did not decrease as observed in WT plants upon BPH infestation. A similar response was observed in *coi1-18* mutants, but in this case, the transcription levels of both *ICS1* and *PAL* as well as the SA concentration increased. These results collectively suggest that JA might participate in the BR-mediated suppression of the SA pathway, reinforcing this antagonistic response. 

Curiously, although BR has been suggested as a negative regulator of innate immunity in plants [132,225,226], it has also been found to positively regulate the defense against the chewing herbivore *Manduca sexta* and the cell-content feeder *Thrips tabaci* [227,228]. These divergent scenarios may be associated with the type of plant tissue affected (root and shoot) and the biotic stressor agent (microbial, virus, insect, necrotrophic, or biotrophic agents); thus, it is very difficult to define a general model of the role of BRs in plant innate immunity and consequently, for BR and JA/SA cross-talk. Furthermore, each plant species, even single plants in the same species, are singular organisms which present different growth–defense trade-offs as a result of resource restriction and these trade-offs are regulated by phytohormone cross-talk in different ways [229].

### 3.7. Brassinosteroids and Strigolactones

Strigolactones (SLs) are a recently discovered group of terpenoid phytohormones that are related to the control of shoot branching [230]. One of the most important signaling components discovered in *Arabidopsis* is *MAX2* (More Axillary Growth Locus 2), which functions to inhibit plant shoot branching [231]. MAX2 constantly interacts with BZR1/BES1 through the PEST domain to mediate their degradation in *Arabidopsis*. The exogenous application of SL induced the degradation of both BR transcription factors mediated by MAX2, and consequently, inhibited shoot branching. Thereby, the interaction between SL and BR may control developmental processes by modulating the MAX2-mediated stability of BZR1 and BES1 [231]. Until now, there has been little data on the SL signaling pathway. It is expected that advances in the research of this new class of phytohormone will more clearly explain the hormonal cross-talk between SL and BR.

## 4. Conclusions and Remarks

As sessile living beings, plants have developed complex mechanisms during their evolution, with phytohormones playing key regulatory roles. The interplay of phytohormones may be used in management and genetic engineering to improve several agricultural traits. In the almost 40 years since the discovery of brassinosteroids as the sixth class of plant hormones, continuous effort has been made to elucidate their role in the multiple aspects of plant physiology. It is known that BRs influence several biological processes, such as growth, protein metabolism, cellular transport and signaling, cell wall biosynthesis, the formation of chromatin and cytoskeleton components, stomatal closure, and environmental responses. Due to the complex network between BRs and other phytohormones and the different physiological effects that this implicates in plant homeostasis, achieving a better understanding of hormonal cross-talk as well as the extensive cross-talk between BRs and other hormones about its role in plant growth and development and responses to stress remains a challenge. This review summarized the previous knowledge about the role of BR cross-talk in plant physiology and compiled the recent findings on these interactions. 

## Abbreviations

ABA	Abscisic Acid
ABI	ABA Insensitive
ACS	ACC-SYNTHASE
ACC	1-aminocyclopropane-1-carboxylicacid
ACO	ACC-OXIDASE ABA Insensitive
AOS2	ALLENE OXIDE SYNTHASE 1
ARF	AUXIN RESPONSE FACTOR
AUX	Auxin
AUX/IAA	AUXIN/INDOLE ACETIC ACID
AuxREs	Auxin-responsive DNA Elements
BAK1	BRI1-ASSOCIATED RECEPTOR KINASE 1
BES1/BZR2	BRI1-EMS SUPPRESSOR 1
BIK1	BOTRYTIS-INDUCED KINASE 1
BIN2	BRASSINOSTEROID-INSENSITIVE 2
BL	Brassinolide
BPH	Brown Planthopper
BRI1	BRASSINOSTEROID INSENSITIVE 1
BRs	Brassinosteroids
BRX	BREVIS RADIX
BRZ	Brassinazole
BSKs	BR-SIGNALING KINASE 1
BSU1	BRI1 SUPPRESSOR 1
bZIP	Basic Leucine-Zipper
BZR1	BRASSINAZOLE RESISTANT 1
CDG1	CONSTITUTIVE DIFFERENTIAL GROWTH 1
ChiP	Chromatin Immunoprecipitation
ChiP-seq	Chromatin Immunoprecipitation-sequencing
CKs	Cytokinins
CKXs	Cytokinin oxidases/dehydrogenases
CPD	CONSTITUTIVE PHOTOMORPHISM AND DWARFISM
DWF4	DWARF 4
EGL3	Enhancer of Glabra 3
ET	Ethylene
Fgl22	Flagellin 22
FLS2	Flg-sensing 2
GAs	Gibberellins
GID1	GIBBERELLIN INSENSITIVE DWARF1
GL3	Glabra 3
HDAC19	HISTONE DEACETYLASE 19
HYD1	HYDRA 1
IAA	Indole Acetic Acid
ICS1	ISOCHORISMATE SYNTHASE 1
IPT	Isopentenyltransferases
JA	Jasmonic Acid
KIB1	KINK SUPPRESSED IN BZR1-1D
LOX1	LIPOXYGENASE 1
LRR	Leucine Rich Repeat
MAMPs	Microbial Associated Molecules Patters
MAPKs	Mitogen Activated Protein Kinases
MAX2	More Axillary Growth Locus 2
MTI	MAMP-triggered Immunity
NPF	NITRATE TRANSPORTER 1/PEPTIDE TRANSPORTER FAMILY
OA	Okadaic Acid
PAL	PHENYLALANINE AMMONIUM LYASE
PAMPs	Pathogen Associated Molecules Patters
PAP1	Anthocyanin Pigment 1
PP2A	PHOSPHATASE 2A
PP2AB’	PP2AB’α and PP2AB’β subunits
PP2C	PHOSPHATASE 2C
QC	Quiescence Center
qRT-PCR	Real-time Quantitative Reverse Transcription-PCR
RLK	Receptor-like kinase
ROS	Reactive Oxygen Species
SA	Salicylic Acid
SAM	Shoot Apical Meristem
SAM²	S-adenosyl methionine
SBI1	SUPPRESSOR OF BRI1
SLs	Strigolactones
SLR1	SLENDER RICE 1
TIR1/AFB	TRANSPORT INHIBITOR RESPONSE1/AUXIN SIGNALING F-BOX
TPL	TOPLESS
WT	Wilt-type
